# The Gene-Lifestyle Interaction on Leptin Sensitivity and Lipid Metabolism in Adults: A Population Based Study

**DOI:** 10.3390/nu9070716

**Published:** 2017-07-07

**Authors:** Harry Freitag Luglio, Dian Caturini Sulistyoningrum, Emy Huriyati, Yi Yi Lee, Wan Abdul Manan Wan Muda

**Affiliations:** 1 Department of Nutrition and Health, Faculty of Medicine, Universitas Gadjah Mada, Yogyakarta 55281, Indonesia; dian.csulis@gmail.com (D.C.S.); emy_huriyati@ugm.ac.id (E.H.); 2 School of Health Sciences, Universiti Sains Malaysia, Gelugor 11800, Malaysia; leeyy.yiyi@gmail.com; 3 Center for Southeast Asian Studies, Kyoto University, Kyoto 606-8501, Japan; wanmanan@gmail.com

**Keywords:** UCP2, leptin, dietary intake, adiposity, obesity

## Abstract

Background: Obesity has been associated with leptin resistance and this might be caused by genetic factors. The aim of this study was to investigate the gene-lifestyle interaction between −866G/A UCP2 (uncoupling protein 2) gene polymorphism, dietary intake and leptin in a population based study. Methods: This is a cross sectional study conducted in adults living at urban area of Yogyakarta, Indonesia. Data of adiposity, lifestyle, triglyceride, high density lipoprotein (HDL) cholesterol, leptin and UCP2 gene polymorphism were obtained in 380 men and female adults. Results: UCP2 gene polymorphism was not significantly associated with adiposity, leptin, triglyceride, HDL cholesterol, dietary intake and physical activity (all *p* > 0.05). Leptin was lower in overweight subjects with AA + GA genotypes than those with GG genotype counterparts (*p* = 0.029). In subjects with AA + GA genotypes there was a negative correlation between leptin concentration (*r* = −0.324; *p* < 0.0001) and total energy intake and this correlation was not seen in GG genotype (*r* = −0.111; *p* = 0.188). Conclusions: In summary, we showed how genetic variation in −866G/A UCP2 affected individual response to leptin production. AA + GA genotype had a better leptin sensitivity shown by its response in dietary intake and body mass index (BMI) and this explained the protective effect of A allele to obesity.

## 1. Introduction

Obesity is an emerging health problem in developing countries [1] because its association with a greater risk to non-communicable diseases such as type 2 diabetes mellitus and cardiovascular diseases [2]. In the last few decades, there has been increasing number of studies that evaluate the etiology of obesity. Majority of biological investigation was focused on the role of pathways in energy metabolism and appetite regulation. Leptin is one of the protein that involved in normal body weight maintenance by regulation of energy homeostasis and food intake [3].

Leptin is a protein that produced mainly by adipose tissue and its production is increased as adipose tissue enlarge [4]. This mechanism is suggested as a homeostasis effort of the body to prevent further weight gain. Nevertheless, leptin appeared to be higher in obese individuals than in normal and this phenomena is suggested as leptin resistance. Leptin resistance is introduced to express the reduced ability to decrease appetite and dietary intake as well as to prevent further weight gain [5]. Until now, it is not clear which factors are responsible for leptin resistance in human. 

There were several studies conducted to evaluate which factors that have been associated with leptin in human. Genetic variation on LEP (leptin) and LEPR (leptin receptor) were previously reported be associated with leptin concentration [6,7,8]. However, to our knowledge, there was no study investigating the role of genetic variation in leptin sensitivity in human. Therefore, we aimed to evaluate whether genetic variation in protein that involved in the leptin signalling pathway that is responsible for leptin resistance.

In this study, we analyzed the association between genetic variation in UCP2 gene and leptin sensitivity in Indonesian population. UCP2 is a subtype of uncoupling proteins which are presented in mitochondria and involved in energy regulation [9,10]. In a population based study, it was shown that UCP2 gene polymorphism was associated with obesity [11,12]. However, it is unclear how UCP2 gene polymorphism was associated with obesity in human. It was previously reported that UCP2 polymorphism was associated with leptin production [13]. Studies in animal trial [14] and human cells [15] showed that leptin was able to induce UCP2 expression. 

Because leptin and UCP2 were involved in energy metabolism, we speculated that there might be an interplay between UCP2 gene polymorphism and leptin sensitivity. Therefore, the aim of this study was to investigate the gene-lifestyle interaction between UCP2, dietary intake and leptin sensitivity in a population based study. Because leptin has an ability to affect appetite and dietary intake, leptin sensitivity was determined by analyzing the correlation between leptin and dietary intake. The correlation between leptin and adiposity also being analyzed separately based on their genotypes to show the differential response towards leptin production. To our knowledge, this is the first study to evaluate the role of UCP2 on leptin sensitivity instead of only leptin production. 

## 2. Methods

### 2.1. Design and Study Population

This was a cross sectional study conducted in the province of Yogyakarta, Indonesia. The subjects were adult men and women with age between 21–56 years old living in the urban area of Yogyakarta city. A total of 380 individuals was selected using a stratified random sampling. Five sub-districts of the city were chosen which represented the area based on population density and subjects were selected randomly. The inclusion criteria were the permanent residents in the area and agree to become subjects of this study by signing the informed consent. The exclusion criteria are those who diagnosed with degenerative diseases such as diabetes, cardiovascular disease, or cancer, pregnant at the moment when the study was done, have a strict diet and have a problem with walking or conducting physical activity in the last 6 months. Ethical clearance was obtained from Ethical Clearance from Medical and Health Research Ethics Committee (MHREC) Faculty of Medicine, Universitas Gadjah Mada, Indonesia (KE/FK/791/EC/2015). This study followed the ethical guidelines of the 1975 Declaration of Helsinki. 

### 2.2. Anthropometric Measurements

Nutritional status was defined by body mass index, which calculated by dividing body weight with the square of height. Body weight was measured using a digital body mass scale (0.01 kg precision, Omron, Osaka, Japan) while the height was measured using microtoise (0.1 cm precision, GEA medical, Jakarta, Indonesia). Definition of overweight was BMI (body mass index) higher than 25 kg/m^2^ while obesity was defined when BMI was higher than 30 kg/m^2^. All anthropometric measurements were done by trained enumerators using calibrated instruments. Waist and hip circumference were measured using a non elastic tape with precision of 0.1 cm (OneMed, Surabaya, Indonesia). Percent body fat was measured using bioelectrical impedance analysis (Omron, Osaka, Japan).

### 2.3. Lifestyle Analysis

Data on lifestyle factors were measured based on dietary intake and physical activity. Data on dietary intake was collected using a semi quantitative food frequency questionnaire (SQ-FFQ) and the analysis was conducted using Nutrisurvey software (EBISpro, Stuttgart, Germany). Data of habitual consumption of food items were collected using SQ-FFQ translated into daily intake. Energy intake was calculated based on total energy (in kcal) that was provided by those food items consumed by subjects. This method was also used to calculate other nutrient consumption, such as protein, fat, and carbohydrate. Database of energy and nutrients content of food items was provided by Nutrisurvey. Data on physical activity was collected using an international physical activity questionnaire (IPAQ). This questionnaire contains information on the intensity and duration for several activities including work/job, transportation, house related work and maintenance, recreation, exercise and leisure-time physical activity. Each activity has a unique MET (metabolic equivalent of task) score, which represent the amount of energy used for a certain type of activity. In order to obtain a great picture of individual physical activity for the whole week, all the activities that has been reported in IPAQ form then calculated into MET-minutes/weeks. The SQ-FFQ and IPAQ were developed and validated before. 

### 2.4. Blood Collection and Biochemistry Analysis

A total of 10 mL subjects blood specimens was collected in Ethylenediaminetetraacetic acid (EDTA)-containing tubes. After collection, blood plasma and buffy coat were separated using a centrifuge. Plasma HDL cholesterol and triglyceride concentration were measured using cholesterol oxidase phenol 4-aminoantipyrine peroxidase (CHOD-PAP) and glycerol phosphate oxidase (GPO) method, respectively (Diasys, Holzheim, Germany). Leptin was measured using enzyme linked immunosorbent assay (DRG, Springfield Township, NJ, USA). 

### 2.5. Genetic Analysis

The DNA sample was isolated from buffy coat using a commercial DNA extraction kit (Favorgen, Pingtung City, Taiwan). UCP2 −866G/A genotyping was done using polymerase chain reaction-restriction fragment length polymorphism (PCR-RFLP) with forward primer: 5′-CACGCTGCTTCTGCCAGGAC-3′ and reverse primer: 5′-AGGCTCAGGAGATGGACCG-3′. PCR conditions are: 8 min of denaturation in 95 °C followed by 35 cycles of 95 °C for 1 min (denaturation), 55 °C for 1 min (annealing), 68 °C for 1 min (extension) and 72 °C for 7 min (final extension). The PCR product then digested using BST UI enzyme digestion. Restriction fragments were resolved on a 3% agarose gel. 

### 2.6. Statistical Analysis

Statistical analysis was conducted in using GraphPad Prism version 5.00 for Windows (GraphPad Software, La Jolla, CA, USA). Subjects were separated into 2 groups: AA + GA and GG. The difference in anthropometric measures, lipid profile, leptin, dietary intake and physical activity between AA + GA and GG genotypes were analyzed using Mann Withney test. Leptin concentration was compared between genotypes and nutritional status using Mann Withney test. A Spearman analysis was done to evaluate the correlation between leptin and dietary intake in each genotype group. A partial correlation analysis was performed to test the correlation between leptin and dietary intake in each genotypes controlled by age, gender and body weight. All statistical analysis was conducted in 2 tail analysis and significance was threshold when *p* < 0.05.

## 3. Results

This is an observational study conducted in the urban area of Yogyakarta, Indonesia. Subjects were 41.7 ± 11.1 years old. To rule out the influence of confounding factors of leptin production such as gender, age and adiposity, the authors calculated the distribution of those characteristics in this study. Men (*n* = 174) and women (*n* = 206) were distributed equally (45.8% and 54.2%, respectively). The distribution of age group were 16.6% for 21–29.9 years old group; 23.2% for 30–39.9 years old group; 30% for 40–49.9 years old group and 30% for 50–56 years old group. In study 26 subjects (6.8%) were underweight (BMI < 18.5 kg/m^2^), 176 subjects (46.3%) were normal weight (BMI 18.5–25 kg/m^2^), 114 subjects (30%) were overweight (BMI 25–30 kg/m^2^) and 64 subjects (16.8%) were obese (BMI > 30 kg/m^2^). The variation of UCP2 gene polymorphism in this study was under Hardy Weinberg Equation (*X^2^* = 0.206, *p* = 0.649). Gender in all UCP2 genotypes were equally distributed: AA male = 26 (48.1%), female = 32 (51.9%); GA male = 89 (48.4%), female = 95 (51.6%), GG male = 59 (41.5%), female = 83 (58.5%). Table 1 shows the comparison of anthropometric, lipid profile, leptin, dietary intake and physical activity between UCP2 genotypes. In this study, we showed that UCP2 gene polymorphism in Indonesian adults was not associated with adiposity, lipid profile and leptin production. There were no differences in energy intake and physical activity between those genotypes. 

**Figure 1:**
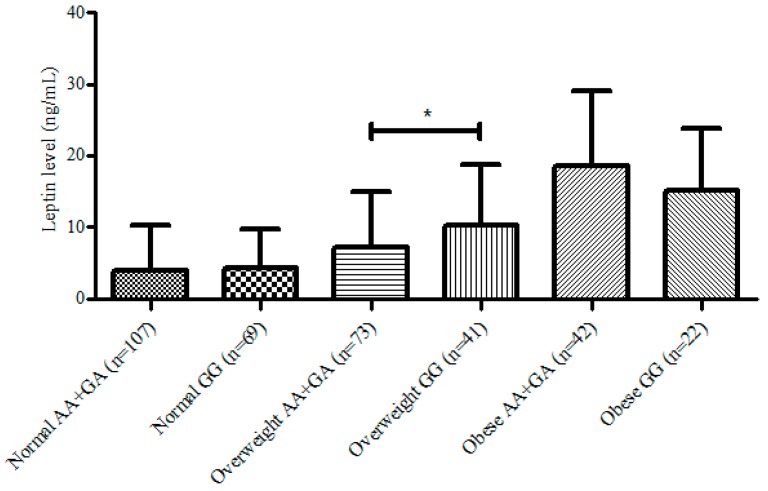
Difference in leptin production across genotypes and nutritional statuses. * Mann Whitney test *p* < 0.05.

Plasma leptin concentration between nutritional statuses were compared (Figure 1). There were no differences in leptin concentration between AA + GA and GG group in normal weight and obese individuals. In subjects with overweight, we showed that leptin concentration in the GG group was significantly higher than those in AA + GA group (*p* = 0.029). 

The correlation between leptin on adiposity, lipid profile and lifestyle factors were analyzed separately depend on UCP2 gene variation. Table 2 shows the correlation between leptin, adiposity and lipid profile. Leptin was correlated with adiposity in both single nucleotide polymorphisms (SNPs). Interestingly, leptin was associated with increased HDL level only in individuals with AA + GA genotypes (*p* = 0.022) and this correlation was not seen in GG genotypes (*p* = 0.822). Table 3 shows the correlation between leptin and lifestyle factors. The correlation between leptin and dietary intake was not seen in GG group. By contrast, leptin was negatively correlated with all dietary intake aspects of individuals in AA + GA group and this correlation is still significant after controlled for age, gender and body weight. In both groups, leptin was not correlated with physical activity.

## 4. Discussions

This study was aimed to elucidate the role of UCP2 gene polymorphism on leptin resistance by taking account subjects dietary intake as a response to leptin production. Leptin is a protein known by its ability to suppress dietary intake by reducing appetite [5]. In this study, we showed the interaction between UCP2 gene polymorphism with dietary intake by the modulation response to leptin. First, leptin was significantly lower in overweight subjects with AA + GA genotypes than those with GG genotype. Second, in subjects with AA+GA genotypes leptin concentration was inversely correlated dietary intake while this correlation was not seen in GG genotype. 

UCP2 is a protein that involved in energy metabolism and the polymorphism of this gene was associated with obesity [11,12,16]. It was previously reported that mRNA expression of UCP2 at the intraperitoneal adipose tissue of obese individuals is lower than their normal counterparts [17]. Interestingly, the study also reported that the −866 genotype of UCP2 gene was associated with intraperitoneal adipose tissue UCP2 mRNA expression [17]. The A allele of −866G/A UCP2 gene polymorphism was associated with higher UCP2 mRNA expression compared to those with the G allele. It was suggested that –866 G/A polymorphism in the UCP2 gene promoter influence exon-8 insertion:deletion transcript ratio and trans-activating effect in human adipocyte cell line [17]. 

In order to test the association between UCP2 gene polymorphism and obesity, subjects were divided into 2 groups: AA + GA and GG genotype. This is based on previous finding which showed that the effect of AA and GA on obesity are similar while GG has a strong effect [17]. In this study, we showed that there were no difference on adiposity, leptin, lipid profile and lifestyle between AA + GA and GG genotypes. 

There was no association between UCP2 gene polymorphism on leptin concentration in all subjects. This result is similar to those found in Italian [18] and Indian [19] population. Because leptin production is directly correlated with adiposity [4], the association between UCP2 and leptin according to subject’s adiposity statuses (normal weight, overweight and obese). Leptin was significantly higher in overweight subjects with GG genotype than those with AA + GA genotypes and this association was not seen in normal weight subjects. This result indicated that subjects in the GG group had less leptin sensitivity compared to those in AA + GA group. It was previously suggested that leptin has the ability to reduce body weight by increasing energy expenditure and lowering appetite, especially when adiposity was increased [20,21]. The lack of leptin efficiency on inducing normal body weight in those who are overweight or obese has been proposed as leptin resistance [22]. We suggest that this phenomena was we showed in overweight subjects with GG genotypes of UCP2 gene. 

Our finding support previous investigation showed that leptin resistance was genetically inherited [23]. Although it has been reported that UCP2 gene polymorphism was associated with leptin concentration [13], the correlation between UCP2 gene polymorphism and leptin sensitivity is still less understood. However, several reports have supported the theory that leptin requires UCP2 to increase metabolism in mitochondria. In vitro, leptin induced up regulation of UCP2 which in turn increased mitochondrial biogenesis and uncoupled respiration [24]. The impact of leptin on uncoupled respiration has also been reported animal trial and this affect adaptive thermogenesis [25]. In addition, genetic polymorphism in uncoupling proteins (UCP2/3) was associated with improvement of mitochondrial function in human [26].

In subjects with AA + GA genotypes there was a negative correlation between leptin concentration and dietary intake while this correlation was not seen in GG genotype. This is confirmed by the correlation with energy providing nutrients such as fat, carbohydrate, and protein intake. Leptin is a protein which produced as a response to increased adiposity and had the ability to reduce appetite and dietary intake. Therefore, we assumed that the negative correlation between leptin and dietary intake showing a normal dietary response to leptin regulation. This response was not seen in subjects with GG genotype, confirming that their leptin sensitivity was affected. The interaction between UCP2 gene polymorphism, dietary intake and obesity was also seen in our previous studies [27,28]. 

In this paper, “leptin resistance” or “lower sensitivity” was exhibited by the interaction between circulating plasma leptin and dietary intake. In scientific publications, leptin has been introduced as product of adipose tissue with the ability to control dietary intake and energy metabolism [3]. And the phrase “leptin resistance” has also been mentioned in many papers as one of parameter in animal trial. However, currently there is no standard for definition of “leptin resistance” in human. Leptin resistance was labelled as signature of high leptin concentration in the human body, but this terminology is rather difficult to apply because leptin concentration is increasing as adipose tissue increases [22]. Therefore the definition of leptin resistance should be based on the interaction between circulating leptin concentration and body response to leptin (for example dietary intake and basal energy expenditure). The limitation of measurement is that dietary intake could be controlled not only by appetite, but also by socio-economic, food availability and other factors. Leptin production is affected by several factors, including gender, age, adiposity, physical exercise, feeding and caloric restriction [29]. Gender, age and adiposity were equally distributed in this study. Physical activity was controlled by excluding those who had problems with walking and conducting normal physical activity. Feeding and caloric restriction was controlled by excluding those is currently following certain weight loss or disease specific diet.

## 5. Conclusions

In summary, we showed that genetic variation in −866G/A UCP2 on individual response to leptin. AA + GA genotype had a better leptin sensitivity shown by its response in dietary intake and BMI. These results also shows the possible explanation on how A allele possess a protective effect to obesity. Further study is needed to study the mechanism on how UCP2 gene polymorphism affects leptin signalling and response in human. Analysis on the correlation between leptin and appetite or basal energy expenditure will be interesting to be done in the future. 

## Figures and Tables

**Table 1:** Anthropometric, biochemical and lifestyle variation between genotypes.

Measurements	Total (*n* = 380)	AA (*n* = 54)	GA (*n* = 184)	GG (*n* = 142)	*p*_AA + GA vs. GG_ *
Age (years)	41.7 ± 11.1 (40.0)	40.8 ± 10.9 (44.0)	42.4 ± 9.8 (42.0)	41.2 ± 12.8 (43.0)	0.523
Anthropometric measurements					
Body weight (kg)	62.3 ± 13.8 (61.4)	62.1 ± 14.4 (61.5)	62.8 ± 13.8 (62.2)	61.7 ± 13.7 (61.2)	0.518
Height (cm)	157.3 ± 9.4 (156.9)	157.2 ± 8.7 (156.2)	157.4 ± 9.9 (157.0)	157.1 ± 9.2 (156.8)	0.748
BMI (kg/m^2^)	25.2 ± 5.3 (24.6)	25.2 ± 5.5 (25.5)	25.3 ± 5.4 (24.6)	25.0 ± 5.1 (24.5)	0.724
Waist circumference (cm)	87.0 ± 13.2 (85.5)	86.1 ± 13.3 (88.4)	86.7 ± 12.8 (84.8)	87.6 ± 13.7 (86.0)	0.469
Hip circumference (cm)	94.3 ± 11.7 (94.0)	94.1 ± 12.3 (94.8)	94.5 ± 11.7 (93.8)	94.2 ± 11.4 (93.8)	0.853
Body fat (%)	28.3 ± 8.8 (28.3)	28.0 ± 8.6 (26.6)	28.2 ± 8.6 (28.0)	28.5 ± 9.1 (29.7)	0.651
Plasma lipid profile					
Triglyceride (mg/dL)	134.7 ± 68.6 (109.0)	133.2 ± 72.2 (110.5)	140.9 ± 77.4 (112.5)	127.4 ± 53.3 (107.0)	0.081
HDL cholesterol (mg/dL)	50.7 ± 44.4 (43.0)	54.5 ± 59.8 (42.5)	52.1 ± 51.3 (43.0)	48.0 ± 23.1 (44.0)	0.322
Plasma leptin (ng/mL)	7.4 ± 8.8 (4.0)	7.1 ± 9.4 (3.5)	7.4 ± 9.2 (4.0)	7.5 ± 8.1 (5.0)	0.871
Dietary intake					
Total energy (kcal)	2043.5 ± 775.6 (1884.5)	2060.1 ± 773.9 (1914.4)	2005.8 ± 761.1 (1833.9)	2086.2 ± 797.6 (2000.3)	0.409
Protein (g)	60.0 ± 29.3 (55.7)	60.6 ± 25.2 (56.8)	57.8 ± 27.3 (54.1)	62.5 ± 33.1 (59.5)	0.195
Fat (g)	53.5 ± 29.7 (47.8)	52.0 ± 25.6 (43.8)	52.6 ± 31.4 (47.4)	55.2 ± 28.9 (48.6)	0.384
Carbohydrate (g)	331.8 ± 131.8 (307.4)	338.0 ± 128.6 (312.9)	325.5 ± 129.2 (294.1)	337.7 ± 136.7 (313.2)	0.502
Physical activity (METS-minute)	5517.2 ± 5666.4 (3689.0)	5269.6 ± 6495.8 (3396.7)	5825.3 ± 5682.6 (4133.8)	5210.0 ± 5316.5 (3554.5)	0.417

BMI: body mass index; HDL: high density lipoprotein; METS: metabolic equivalent of task * Data is shown as mean ± standard deviation (median) * Independent *t*-test analysis.

**Table 2:** Correlation between leptin and adiposity within UCP2 genotypes.

	AA + GA (*n* = 238)		GG (*n* = 142)	
	***r***	***p***	***r***	***p***
Age	−0.021	0.742	0.071	0.400
Body weight	0.415	<0.0001	0.321	<0.0001
Height	−0.302	<0.0001	−0.365	<0.0001
Body mass index	0.640	<0.0001	0.567	<0.0001
Waist circumference	0.475	<0.0001	0.497	<0.0001
Hip circumference	0.629	<0.0001	0.503	<0.0001
Body fat (%)	0.709	<0.0001	0.626	<0.0001
Triglyceride	0.002	0.974	−0.087	0.303
HDL cholesterol	0.150	0.022	0.019	0.822

**Table 3:** Correlation between leptin and lifestyle factors within UCP2 genotypes.

	AA + GA (*n* = 238)			GG (*n* = 142)		
	***r***	***p*** ***^a^***	***p*** ***^c^***	***r***	***p*** ***^a^***	***p*** ***^c^***
Energy intake	−0.324	<0.0001	0.004	−0.111	0.188	0.148
Protein intake	−0.268	<0.0001	0.001	−0.065	0.439	0.104
Fat intake	−0.186	0.004	0.003	0.064	0.450	0.779
Carbohydrate	−0.323	<0.0001	0.031	−0.158	0.060	0.140
Total physical activity	−0.005	0.942	0.101	−0.045	0.599	0.528

*p* ^a^ = *p*-value for Pearson correlation analysis *p* ^c^ *=* *p*-value for partial correlation analysis controlled for age, gender and body weight.
